# Bioinspired trajectory modulation for effective slip control in robot manipulation

**DOI:** 10.1038/s42256-025-01062-2

**Published:** 2025-07-22

**Authors:** Kiyanoush Nazari, Willow Mandil, Marco Santello, Seongjun Park, Amir Ghalamzan-E

**Affiliations:** 1 School of Computer Science and LIAT, University of Lincoln, Lincoln, UK; 2 Cambridge Research Laboratory, Toshiba Europe, Cambridge, UK; 3 School of Biological and Health Systems Engineering, Arizona State University, Tempe, AZ USA; 4 Department of Bio and Brain Engineering, Korea Advanced Institute of Science and Technology (KAIST), Daejeon, Republic of Korea; 5 School of Computer Science and Electronic Engineering, University of Surrey, Guildford, UK

**Keywords:** Mathematics and computing, Electrical and electronic engineering

## Abstract

Ensuring a stable grasp during robotic manipulation is essential for dexterous and reliable performance. Traditionally, slip control has relied on grip force modulation. Here we show that trajectory modulation provides an effective alternative for slip prevention in certain robotic manipulation tasks. We develop and compare a slip control policy based on trajectory modulation with a conventional grip-force-based approach. Our results demonstrate that trajectory modulation can significantly outperform grip force control in specific scenarios, highlighting its potential as a robust slip control strategy. Furthermore, we show that, similar to humans, incorporating a data-driven action-conditioned forward model within a model predictive control framework is key to optimizing trajectory modulation for slip prevention. These findings introduce a predictive control framework leveraging trajectory adaptation, offering a new perspective on slip mitigation. This approach enhances grasp stability in dynamic and unstructured environments, improving the adaptability of robotic systems across various applications.

## Abstract (web summary)

When a robot grips and moves a delicate object, it can slip from grasp. Instead of gripping the object with more force, a method is proposed here to move the object in a way that prevents slippage.

## Main

Slippage of objects (we call it ‘slip’) during robotic manipulation tasks can result in instability and task failure^1,2^. Robotic systems are still far behind human-level dexterity in handling slip^3^. Improving slip controllers can be attributed to enhancing tactile sensors^4–6^ for reliable slip detection^7–9^ and intelligent control strategies for slip prevention^10^. In this study, we focus on the latter and present a bioinspired^11^ approach for slip control. Current slip controllers primarily rely on increasing grip force in response to detected slip events^10^. However, it is still unclear whether grip force modulation is the only means of slip control. Inspired by the inherent dynamics of slip events indicating rapid adjustments in motion trajectories can stabilize the physical interaction^12,13^ and findings in ref. ^11^ showing humans deploy hand acceleration adaptation besides grip force modulation for slip prevention, we explore the effectiveness of trajectory modulation as an alternative control policy in robotic pick-and-place tasks where grip force control is not always possible or effective. The code can be found at https://github.com/imanlab/bgf, and our dataset and other information are available at https://proactive-control.github.io/.

Grip force control in robotics (based on detected^14–19^ or predicted^10^ slip instances) is inspired by neurophysiological studies on humans’ control policies (for example, refs. ^20–22^). These studies have shown that humans optimally adjust the grip force to be slightly larger than the required amount for preventing object slip. However, grip force control may not be effective or possible in many robotic scenarios, for example, when the maximum grip force is already applied^23^ or when handling delicate objects^24^. Moreover, many robotic architectures do not permit real-time grip force control, for example, the Franka Emika arm^25^. Hence, our hypothesis involves a robot controller using hand trajectory modulation that can be as effective in preventing object slip as a controller using grip force modulation.

**Fig. 1:**
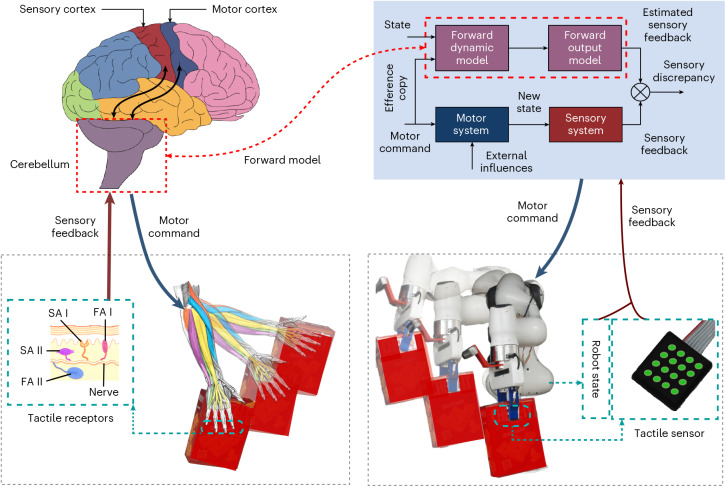
Tactile forward model in sensorimotor loop. Block diagram^50^ illustrating the predictive control architecture in humans based on the internal forward models that are learnt in the cerebellum of the human brain^52,53^, as a design motivation for our proposed proactive controller. The forward model predicts future sensory states based on the current state and a copy of potential motor commands (efference copy) to enable predictive movements without relying on delayed sensory feedback^54,55^. The internal representation in the cerebellum is learnt via neuronal connections with the sensory and motor cortices in the cerebral cortex.

We introduce a predictive trajectory modulation approach for slip control, which dynamically adjusts planned motions in real time to prevent anticipated slippage during robotic manipulation tasks, with supplementary insights from human studies^11^. Although our results show that reactive trajectory modulation reduces slip instances, it is suggested that humans’ dexterity is owed to a predictive control system relying on internal forward models^26,27^. Forward models (Fig. 1) are thought to enable the prediction of sensory consequences of motor actions and allow bypassing unavoidable delays due to latency associated with sensorimotor loops^27–30^, whereas the motor cortex defines optimal actions to achieve the task goal^31^. Hence, our next hypothesis is to test whether a predictive controller can yield better performance in slip control. Learning a forward model in physical robot interaction with the high-dimensional state space^32,33^ is challenging and has limited the robotic systems to benefit from predictive controllers in cases such as slip prevention.

We, therefore, proposed a six-dimensional proactive control for trajectory modulation to avoid slip, relying on a learned tactile forward model that predicts slip instances within a prediction horizon.

A predictive controller adjusts the reference trajectory based on a predicted slip signal to minimize the likelihood of future slips. This extends our previous one-dimensional controller^34^ to a six-dimensional velocity controller and leverages our state-of-the-art action-conditioned tactile prediction (ACTP) model^35^ for slip prediction. Proactive control closes the control loop with the predicted system states and outperforms current reactive grip force controllers by achieving a smaller likelihood of slip control failure. Proactive slip control also benefits from a slip classification on the ACTP prediction signals, making it superior to that of a simple long short-term memory (LSTM)-based classification^34^, contributing to the improved performance of the proactive controller.

We conducted a series of experiments using the Franka Emika arm to demonstrate the effectiveness of reference trajectory modulation. We implemented and tested both reactive and proactive controls. Our experiments include a variety of objects, trajectory classes and robotic manipulation tasks, in which slip may cause task failures. Our results show that trajectory modulation is effective in controlling slip where grip force control is not either possible or sufficiently effective in robotic manipulation. We evaluated the generalization capabilities of proactive control for trajectory modulation. We observed the controller generalizes well to unseen object classes, robot trajectories and motion start/end poses.

Our contribution is manifold: we propose a robotic slip control approach that utilizes reference trajectory modulation. Inspired by the proactive control strategy used by humans^36,11^, we introduce a proactive control framework that uses a forward model to predict slip incidents and ensure task success in physically interactive scenarios. The proposed approach offers a substantial improvement over current slip control methods.

## Results

We aimed to explore the role of trajectory modulation in slip control. Our predictive control strategy uses a forward model, in contrast to the reactive grip force control that is commonly used in robotics, to predict the consequence of its action and select future trajectory points, yielding no slip during manipulative movements. Our findings in our previous study^11^ indicate that humans rely on trajectory modulation, when grip force control is insufficient, for slip control. This insight motivated the development of our proactive control policy that modulates trajectories to prevent slip. Ref. ^11^ demonstrates that participants use hand acceleration modulation as a strategy to minimize task completion time and prevent slip occurrences. Building on this insight, we have developed a slip control approach for robotic manipulation. This innovative method involves optimizing the robot’s predetermined trajectory through the application of its tactile forward model, directly addressing our second hypothesis and representing a notable advancement in the field of robotic slip control.

### Trajectory modulation for object slip control

#### Hypothesis

**Fig. 2:**
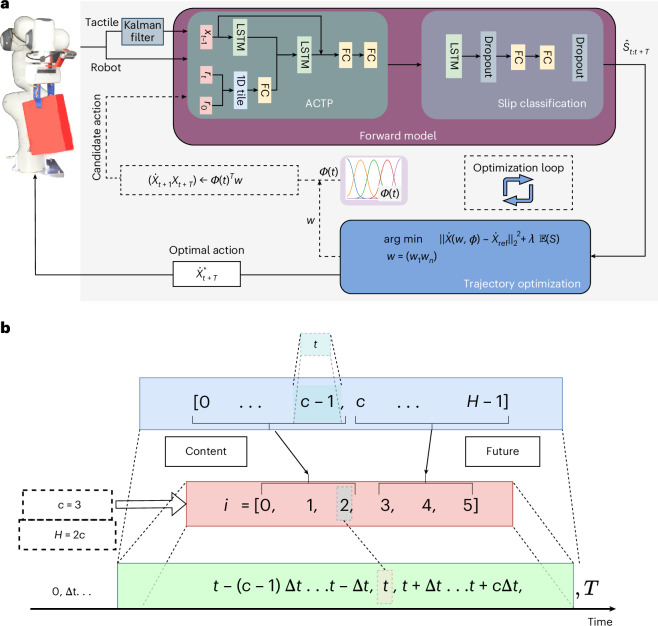
Proactive slip control architecture. **a**, Proactive control system, consisting of ACTP, slip classifier and predictive controller. The system aims to minimize the likelihood of future slip by minimum deviations from a reference trajectory. At each optimization iteration, the objective function is evaluated by passing the constructed robot actions by trajectory weights to the tactile forward and slip classification models. The signals shown by solid lines are received/commanded in real time (20 Hz), and the ones with dashed lines are in the optimization loop (100 Hz). 1D, one dimensional. **b**, At each time *t*, a context and prediction window of size *c* is used by the ACTP model, where the entire context and prediction window has *H* = 2 × *c* time samples; hence, the context and prediction times are {*t* − (*c* − 1)Δ*t*…*t* − Δ*t*, *t*} and {*t* + Δ*t*…*t* + *c*Δ*t*} assuming a fixed sampling frequency Δ*t* in the dataset. For *c* = 3, the context is {*t* − 2Δ*t*, *t* − Δ*t*, *t*} and future frames {*t* + Δ*t*, *t* + 2Δ*t*, *t* + 3Δ*t*} and the corresponding index in our prediction model is *i* = {0, 1…5}. FC, fully connected layers.

**Fig. 3:**
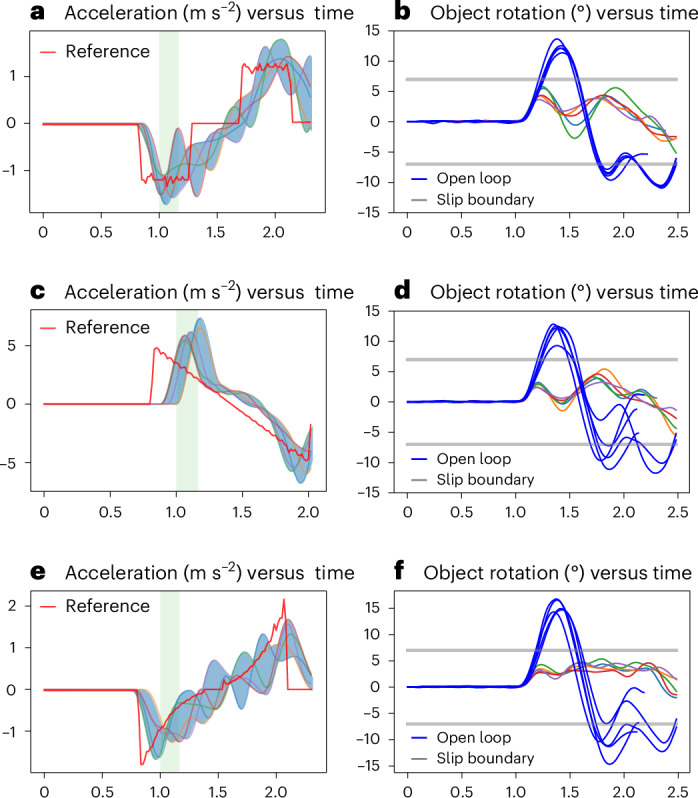
Trajectory modulation for slip control results. Results obtained using our proactive controller with trajectory modulation for slip control in a linear pick-and-place task. **a**,**c**,**e**, Reference Cartesian robot trajectory/acceleration (red line) for trapezoidal (**a**), cubic (**c**) and quintic (**e**) trajectories. **b**,**d**,**f**, Corresponding object rotations around the grip point, with the blue lines representing the object’s rotation. The variation in object rotation for the same reference trajectory is attributed to nonlinear interactions between the object and the gripper. The other coloured lines represent the trajectories generated by the proactive closed-loop slip controller (left column) and the corresponding rotation angles (right column). The proactive controller aims to maintain the object rotation below 6°. A visual analogy can be drawn between these results and those of human slip control behaviour^11^.

A robot controller using reference trajectory modulation can be effective in slip control: we attached a pair of uSkin^37^ tactile sensors to Franka Emika robotic arm’s fingers for closing the loop for trajectory modulation. Our proactive controller involves lifting an object from a top grasp pose by a parallel gripper and moving it to the target pose by modulating a given reference trajectory. Object slip is predicted in real time within a specified prediction window using a tactile forward model. The trajectory optimization pipeline (Fig. 2) then leverages these predicted slip values to learn how to dynamically adjust the Cartesian reference trajectory, thereby minimizing the likelihood of future slip events. If the tactile forward model does not predict a positive slip event during the trial, the predefined reference trajectory will be executed without modification. Figure 3 shows the robot Cartesian-space acceleration for a linear movement from (0.4, –0.3, 0.3) to (0.1, 0.25, 0.3) in the robot base frame (Table 1). This result obtained with the Domino object, which is a sample object from our test objects set (see the list of ten objects used for training and three objects used for testing the tactile forward model, namely, the train object set and test object set; Table 2). The object’s in-hand pose is measured using a wrist-mounted Intel RealSense camera and a visual marker attached to the object. The threshold for switching the contact state from stick to rotational slip was chosen as 6°. The small deformation of the tactile sensor taxels allowed <6° object rotation without slip.

**Table 1:** Comparison of proactive control by trajectory modulation with grip force control as the baseline model on different reference trajectories and classes of test motions averaged over three test objects

Control	Test motion	Reference trajectory	RTS*↓*	MOR (°)*↓*	DRT (cm s^−1^)	ROV*↓*	ET*↓*
**Grip force slip control (reactive)**	TM-1	Trapezoid	29.32 ± 5	15.45 ± 1	–	–	–
		Cubic	26.11 ± 4	8.68 ± 0.8	–	–	–
		Quintic	23.52 ± 2	8.22 ± 1.2	–	–	–
	TM-2	Trapezoid	31.08 ± 8	14.23 ± 1.1	–	–	–
		Cubic	26.04 ± 4	9.43 ± 0.3	–	–	–
		Quintic	25.25 ± 5	13.43 ± 1.8	–	–	–
	TM-3	Trapezoid	28.64 ± 4	13.65 ± 0.9	–	–	–
		Cubic	30.41 ± 3	11.43 ± 1.1	–	–	–
		Quintic	26.00 ± 3	9.10 ± 1.3	–	–	–
	–	**Mean**	27.37 ± 2	11.51 ± 2.5	–	–	–
**Trajectory modulation slip control (proactive)**	TM-1	Trapezoid	3.6 ± 2.0	7.44 ± 1.9	1.62 ± 0.1	0.81 ± 0.1	0.10 ± 0.03
		Cubic	1.8 ± 3.6	6.66 ± 1.5	1.64 ± 0.1	0.62 ± 0.1	0.10 ± 0.03
		Quintic	**1.0** ± **2.0**	**5.60** ± **1.2**	1.71 ± 0.2	0.47 ± 0.2	0.10 ± 0.02
	TM-2	Trapezoid	8.5 ± 3.3	6.92 ± 1.1	1.38 ± 0.1	0.68 ± 0.2	0.10 ± 0
		Cubic	**0** ± **0**	**5.81** ± **0.9**	1.67 ± 0.1	0.35 ± 0.1	0.10 ± 0.03
		Quintic	2.0 ± 2.5	6.74 ± 0.9	1.69 ± 0.1	0.51 ± 0.2	0.10 ± 0.02
	TM-3	Trapezoid	5.3 ± 3.0	6.04 ± 1.3	1.60 ± 0.1	0.71 ± 0.2	0.10 ± 0.03
		Cubic	4.6 ± 4.9	7.73 ± 2.2	1.30 ± 0.1	0.70 ± 0.2	0.11 ± 0.03
		Quintic	**1.9** ± **1.5**	**5.44** ± **1.4**	1.74 ± 0.2	0.54 ± 0.2	0.11 ± 0.03
	–	**Mean**	3.1 ± 1.5	6.48 ± 1.7	1.59 ± 0.1	0.59 ± 0.1	0.10 ± 0

The test motions (TM-1, TM-2 and TM-3) are three common pick-and-place tasks. The definitions of the RTS, MOR, DRT and ROV metrics are provided in Table 2. ET, execution time in milliseconds indicating the computation cost. *↓* or *↑* shows a lower or higher value is better, respectively. The best performance metrics for RTS and MOR for each test motion are highlighted in bold. The mean and standard deviation values are computed over five repeated executions of the same manipulation trial.

**Table 2:** Proactive controller performance on the object transport task with trapezoidal velocity for individual train and test set objects

Test object			RTS*↓*	MOR (°)*↓*	DRT (cm s^−1^)	ROV*↓*
Train	01	BreadSticks	**0** ± **0**	4.87 ± 0.27	1.60 ± 0.14	**0.33** ± **0.08**
	02	CORNFLOUR	2.8 ± 2.48	7.33 ± 0.69	1.59 ± 0.21	0.51 ± 0.19
	03	Ravita THINS	**0** ± **0**	4.62 ± 0.44	1.69 ± 0.08	0.82 ± 0.24
	04	PUFF pastry	8.2 ± 7.41	7.11 ± 1.65	1.47 ± 0.15	0.36 ± 0.12
	05	GRISSINI	5.0 ± 5.93	7.00 ± 1.41	1.70 ± 0.18	0.44 ± 0.16
	06	Batter Mix	**0** ± **0**	5.03 ± 1.21	1.59 ± 0.12	0.59 ± 0.17
	07	Jaffa cake	7.8 ± 6.73	8.02 ± 1.20	1.69 ± 0.15	0.82 ± 0.25
	08	KLEENEX	5.0 ± 6.13	7.62 ± 1.45	1.42 ± 0.06	0.48 ± 0.17
	09	CHEEZEIT	**0** ± **0**	**2.62** ± **0.46**	1.61 ± 0.16	0.49 ± 0.17
	10	RICE	**0** ± **0**	4.54 ± 0.90	1.43 ± 0.11	0.76 ± 0.19
	**Mean**		3.22 ± 3.09	5.98 ± 1.73	1.57 ± 0.10	0.58 ± 0.01
Test	11	CUPa SOUP	**0** ± **0**	**3.75** ± **1.17**	1.52 ± 0.12	**0.53** ± **0.17**
	12	Carrs	10.4 ± 6.08	8.89 ± 1.75	1.54 ± 0.16	0.71 ± 0.09
	13	Domino	**0** ± **0**	5.09 ± 0.73	1.69 ± 0.09	0.66 ± 0.21
	**Mean**		3.46 ± 4.90	5.91 ± 2.17	1.58 ± 0.07	0.63 ± 0.02

RTS, the number of time steps in which object rotation is >6°; the higher the number is, the higher probability that the task fails; MOR, maximum amount of object rotation; DRT, the sum of Euclidean distances between the reference and adapted trajectories over the task execution time; ROV, the value of the objective function after optimization; *↓* or *↑* show lower or higher values are better, respectively. Full slip prevention trials (for example, RTS = 0) and the best performance metrics for MOR and ROV across the train and test set objects are highlighted in bold. The mean and standard deviation values are computed over five repeated executions of the same manipulation trial.

We show the results of trajectory modulation for three reference velocity trajectories (Fig. 3): trapezoid (panel a), cubic (panel c) and quintic (panel e), and the object rotation achieved by the proactive controller in panels b, d and f, respectively. For each test case, we repeated each task execution five times. Variations are due to the nonlinear nature of the slip, variability in initial contact and stochastic nature of the forward model used in our proactive controller. The onset of the predicted slip is shown with a green-shaded band in Fig. 3a,c,e. This shows that the proactive controller effectively controls slips regardless of the onset time and nature. Figure 3b,d,f shows the object rotation (blue line) without the proactive controller where the reference trajectory is executed without modulation.

The data shown in Fig. 3 demonstrate that the object rotations obtained by proactive control are significantly smaller than those obtained with the open-loop controller and remain within the set slip boundaries. These achievements can be attributed to the deviations of the generated trajectories from the reference trajectory. Proactive control can effectively minimize slip incidents by modulating the robot’s trajectory despite a constant grip force applied, which is within a safety margin for forming a stable grasp and lifting the object. These are in agreement with the human slip control strategy presented in ref. ^11^.

The rows showing the mean value for DRT (Table 2) explain that the deviation from the reference trajectory remains comparatively close between the train and test objects, illustrating the effectiveness of our proposed approach in generalizing to unseen objects during training. Nonetheless, the ROV has an 8% increase for the test objects compared with the train set objects, showing that a reach object set (representing a variety of object classes) can enhance the performance.

We also tested the proactive control in three classes of manipulative movements, each with a varying number of degrees of freedom (DOFs) and quantitatively compared its results with a baseline slip control model, that is, a reactive grip force control with an MHZ2 SMC gripper. For each motion class, we fix a few DOFs in Cartesian space and generate a reference trajectory for the remaining DOFs to set the robot’s velocity. This allows us to examine the relationship between specific movements and slip instances. We focus on test movements relevant to common pick-and-place tasks, including linear motions in a horizontal plane (TM-1), motions in a horizontal plane with wrist rotation (TM-2) and lifting motions in a vertical plane by moving an object up and down (TM-3).

As a baseline, we use a reactive (grip force) control, which is the common approach in the literature, using the MHZ2 SMC gripper to modulate the grip force in real time. By attaching uSkin sensors to the fingers, we created a setup that is comparable with one with the default Franka Emika gripper, which cannot be controlled in real time. The grip force slip control method is a reactive control system that tightens the grip whenever a positive slip signal is detected. We repeated each test case ten times and report the mean and standard deviation for each performance metric. Table 1 presents the average performance of reactive control for test objects set on various reference trajectories and classes of motion. Both RTS and MOR values in Table 1 indicate that the trapezoid reference trajectory is the most challenging for controlling the slip in all three classes of test motions. This is due to the jerk in the phase of switching from linear to constant velocity and vice versa. Although the quintic reference has the lowest RTS in all test motions, the cubic reference has the smallest MOR in TM-2. This table shows that the average MOR for reactive grip force control is 11.51°, which is 5.51° larger than the slip threshold. We conduct the same test cases for our proposed proactive controller and present the results in Table 1. On the basis of RTS and MOR values, similar to reactive control, the trapezoid reference trajectory is the most challenging for proactive control for slip avoidance. However, proactive control showed notable improvement for both metrics in each test motion with all reference trajectories compared with reactive control. Although the quintic reference has the lowest RTS and MOR in TM-1 and TM-3, in TM-2, the cubic reference has the best RTS and MOR values. The mean value for MOR for proactive control is 6.48°, which has 82% improvement with respect to reactive control. The mean RTS value for proactive control is also 780% smaller than the mean RTS for reactive control. We also present three other metrics specific to proactive control for its optimization performance evaluation. ET shows the execution time in seconds. Table 1 shows that the deviation from the quintic reference is the largest for all classes of test motions. ROV values agree with RTS and are indicative of the convergence of the optimization loop. ET is close for all three classes of test motions.

These results elucidate our hypothesis, proving that slip control with robot trajectory modulation can be significantly more effective than the common grip force slip controllers in certain scenarios. Furthermore, the use of the tactile forward model for trajectory modulation increases the slip controllers’ reaction time, resulting in better performance than the reactive grip force modulation for slip control.

#### Slip control generalization to novel trajectory start and end poses

We examined the impact of the robot movements within the robot’s workspace on the model’s performance by testing the model to pick the objects and place them with start and end points unseen during training. The coordinates are as follows: the original (*x*, *y*, *z*)_start_ = (0.4, –0.3, 0.3) and (*x*, *y*, *z*)_goal_ = (0.1, 0.25, 0.3), and new points (*x*, *y*, *z*)_start_ = (0.3, 0.4, 0.3) and(*x*, *y*, *z*)_goal_ = (0.15, –0.2, 0.3) in the robot base frame, respectively. This helps us understand how much the variance in the task-space start and end poses could affect the model’s performance. Table 3 presents the mean performance of the controllers with ten repetitions for the three test objects with new start and goal points. A comparison of the results in Table 1 and Table 3 illustrates that the controller is robust to changes in the motion zones in the task space and it can effectively adapt to different start and end points within the robot’s workspace.

**Table 3:** Proactive controller’s performances with novel Cartesian start and goal points for TM-1 with trapezoid reference trajectory

Test motion	Reference trajectory	RTS*↓*	MOR*↓*	DRT	ROV*↓*
TM-1	Trapezoidal	6.0 ± 1.51	7.91 ± 0.35	1.61 ± 0.16	0.85 ± 0.07
	Cubic	4.8 ± 2.93	6.77 ± 2.67	1.71 ± 0.16	0.56 ± 0.08
	Quintic	**3.9** ± **1.81**	**6.07** ± **0.19**	1.83 ± 0.35	0.41 ± 0.10
TM-2	Trapezoidal	5.2 ± 3.61	7.25 ± 1.26	1.49 ± 0.17	0.71 ± 0.19
	Cubic	**1.6** ± **0.74**	**6.09** ± **1.11**	1.83 ± 0.06	0.44 ± 0.21
	Quintic	2.2 ± 0.90	6.54 ± 0.56	1.72 ± 0.12	0.46 ± 0.11
TM-3	Trapezoidal	5.9 ± 1.46	7.64 ± 0.50	1.58 ± 0.31	0.78 ± 0.14
	Cubic	3.0 ± 1.3	6.94 ± 0.96	1.53 ± 0.23	0.58 ± 0.09
	Quintic	**2.9** ± **2.5**	**6.03** ± **1.52**	1.80 ± 0.19	0.51 ± 0.17
	Mean	3.94 ± 1.52	6.80 ± 0.65	1.67 ± 0.12	0.59 ± 0.14

The scores are the average of three test objects. The definitions of the RTS, MOR, DRT and ROV metrics are provided in Table 2. The best performance metrics for RTS and MOR for each test motion are highlighted in bold. The mean and standard deviation values are computed over five repeated executions of the same manipulation trial.

#### Hypothesis

Proactive slip control can outperform reactive slip control with trajectory modulation: we show the advantages of proactive trajectory modulation compared with reactive trajectory modulation in a pick-and-place task. Although the former utilizes the tactile forward model consisting of ACTP and slip classification model for slip prediction, the latter uses only a slip classification model to detect slip at the current time. We use TM-1 and Domino as the comparison task motion and object (Table 1), respectively. In the ten trials of the test, the proactive trajectory modulation significantly reduced the object’s in-hand rotation (35% reduction in MOR) and the number of slip instances (63% reduction in RTS) compared with the reactive trajectory modulation. The proactive approach starts the trajectory modulation before a real slip incident occurs, whereas the reactive method starts the modulation of the reference trajectory after observing a slip instance. This helped the proactive approach result in better slip control performance. This observation addresses our third hypothesis, which states that proactive trajectory modulation is more effective in slip control than reactive trajectory modulation.

## Discussion

Our research investigated the slip control mechanisms during manipulation, revealing that trajectory modulation, not just grip force, is crucial in slip control, especially during long-range movements. This is in line with findings in human slip control^11^. We proposed an approach that uses predictive controllers, to enhance robot interaction with complex environments. Traditional reactive control methods are limited by their reliance on the observed states, whereas our proactive controller uses predicted slip states to control object slip during object transport tasks. Our method demonstrated substantial improvements in slip control without requiring increased grip force, outperforming traditional grip force control methods. It proved effective across various trajectories, objects and motions, making it versatile and adaptable.

Proactive control offers broader benefits to robotics by enhancing the robots’ ability to operate in unstructured environments, potentially revolutionizing robotic deployment. However, future work should address limitations such as the controller’s neglect of prediction errors, reliance on offline training, limited object set and computational complexity. Furthermore, extending our approach beyond tasks with predefined reference trajectories would further improve its applicability.

## Conclusion

In conclusion, we have tested two hypotheses for designing a controller for robot physical interaction in the context of slip control. First, these results were instrumental in proposing our trajectory modulation slip control approach, which offers a promising solution to cope with complex interactive tasks and dynamics. Second, by integrating an action-conditioned forward model into a predictive control architecture to modulate the reference trajectory, our approach allows robots to use the predicted slip events and adjust their trajectory in real time to avoid those cases in contrast to reactive control policies that activate after the slip occurrence. Our results show a substantial reduction in the likelihood of task failure and an increase in the robot’s success rate compared with traditional reactive control policies. Using the data-driven forward model improved the slip state estimation and slip controller performance. We believe that our approach has notable potential in a variety of industrial and service robotic applications, and our work opens up new opportunities to bring robots into our daily life. We hope that our findings will inspire future research in this area and further advance the field of robotics.

## Methods

Here we delve into the implementation of proactive trajectory modulation as a means of slip control, which addresses our first hypothesis. We present the methodology and experimental setup used to examine the effectiveness of trajectory modulation in preventing slip incidents during robotic manipulation tasks. Furthermore, we introduce the concept of incorporating a forward model in proactive control, an approach aimed at slip avoidance, as per our second research hypothesis. We outline the key aspects of proactive control, its implementation and the experimental framework used to evaluate its performance in slip prevention.

### Experimental setup

Traditional parallel jaw grippers are still widely used in many robotic manipulation tasks, such as bin picking ^38^. Usually, a motion planning module generates a reference trajectory for the robot by minimizing, for example, time or jerk before motion execution. Then, the robot executes them in an open-loop manner with respect to task failure/success due to a slip in real time. This limits the robot’s success, and solutions in the literature suggest increased grip force on the fly, which may not be feasible in many cases^39–41^. We address this issue using our data-driven predictive control concept, referred to as proactive control.

First, we collected a dataset of open-loop trajectory executions in which the robot manipulates an object included in our train objects set shown in Supplementary Table 2. We equipped the fingers of a Franka Emika robotic arm with a pair of 4 × 4 uSkin tactile sensors and performed moving tasks with multiple objects. The uSkin tactile sensor has 16 sensing points (taxels) that measure triaxial forces, including shear *x*, shear *y* and normal *z* (Fig. 1). To create various types of reference trajectory, we used two main motion strategies during data collection: (1) kinaesthetic robot motions, where a human user performed moving tasks with qualitative fast and slow motions; (2) automatic robot motions, where trapezoidal reference trajectories with various acceleration/deceleration values were sent as a reference Cartesian velocity. A D435i camera manufactured by Intel RealSense mounted on the robot’s wrist measures the pose of an ArUco marker attached to the top of the object. The pose data are then post-processed to create binary slip labels based on the object’s in-hand displacement.

#### ACTP

In this work, tactile data are referred to as frames, representing single time-step readings of the respective sensing modality. Given (1) a set of context frames **f**_0:*c*−1_ = {**f**_0_…**f**_*c*−1_}, which are the previously observed frames with a context sequence length of *c*, and (2) a prediction horizon of length *H* – *c* (that is, the number of future frames to predict); here we assume equal context and prediction window size, that is, *H* = 2*c*, where ${i}_{c}\in \{0,\ldots ,c-1\}\in {{\mathbb{Z}}}^{c}$, ${i}_{p}\in \{c,\ldots ,H-1\}\in {{\mathbb{Z}}}^{c}$ and $i\in \{0,\ldots ,c-1\}\cup \{c,\ldots ,H-1\}\in {{\mathbb{Z}}}^{H}$ (Fig. 3b shows details of the samples in the context and prediction horizons). Although here we show the formulation for one time step **f**(*t*), generalization for training for the entire trajectory, that is, *t* = 0:*T*, is straightforward. A tactile prediction model can be defined as **f** (we use *_i:*i*+*n*_ = {*_i_, *_i+1_…*_i+*n*_}; Fig. 3b) to denote variables representing the set of frames and $\hat{{\bf{f}}}$ to denote the corresponding predicted values:

1 $${\hat{{\bf{f}}}}_{c:H-1}={\mathcal{F}}\left({{\bf{f}}}_{0:c-1}\right),$$

where ${\hat{{\bf{f}}}}_{c:H-1}$ represents a set of predicted frames ${\hat{{\bf{f}}}}_{i}\in {{\mathbb{R}}}^{48}$ (tactile images). The goal is to optimize equation (2), for each time step in the prediction horizon counted by *i*_p_:

2 $$\min \mathop{\sum }\limits_{i=c}^{H-1}{\mathcal{D}}\left({\hat{{\bf{f}}}}_{i},{{\bf{f}}}_{i}\right),$$

where ${\mathcal{D}}$ denotes the loss function in the tactile reading space or pixel space, such as ${{\mathcal{L}}}_{1}$ or ${{\mathcal{L}}}_{2}$, measuring the difference between the predicted and observed frames.

In a physical robot interaction, we aim to develop a cause–effect understanding of the robot’s actions. Thus, we condition the prediction model on the past context frames **f**_0:*c*−1_, the past robot trajectory **x**_0:*c*−1_ and planned robot actions **a**_*c*−1:*H*−2_ to output future frames ${\hat{{\bf{f}}}}_{c:H-1}$ (which are known in our physical robot interaction datasets but unknown during the inference time). Here ${{\bf{x}}}_{{i}_{c}}\in {{\mathbb{R}}}^{6}$ represents the robot trajectory at past steps, and ${{\bf{a}}}_{{i}_{p}}\in {{\mathbb{R}}}^{6}$ represents the planned future robot actions at time *t*. The model assumes a known and nearly constant sampling frequency (typically around 10 Hz with less than 10% variance), and we work with discrete values. The prediction model can be expressed as

3 $${\hat{{\bf{f}}}}_{c:H-1}={\mathcal{F}}\left({{\bf{f}}}_{0:c-1},{{\bf{x}}}_{0:c-1},{{\bf{a}}}_{c-1:H-2}\right),$$

where **f** denotes tactile frames. In model predictive control, which commonly uses forward prediction models like those described here, future robot actions are considered as a batch of candidate actions. The optimal action is selected by a discriminator based on the most desirable predicted tactile frames^34,42^.

Our model aims to maximize the $$p\left(\right.{\hat{{\bf{f}}}}_{c:H-1}| {{\bf{f}}}_{0:c-1},$$$$\{{{\bf{x}}}_{0:c-1},{{\bf{a}}}_{c-1:H-2}\},$$ $$\left.{{\bf{z}}}_{0:c}\right)$$ to predict tactile frame $${\hat{{\bf{f}}}}_{c:T}$$ by applying stochastic assumption to the prediction model. Our objective is to sample from

4 $$p\left({\hat{{\bf{f}}}}_{c:H-1}| {{\bf{f}}}_{0:c-1},\{{{\bf{x}}}_{0:c-1},{{\bf{a}}}_{c-1:H-2}\}\right).$$

The network can then be trained with the frame prediction model by maximizing equation (4).

5 $$\begin{array}{ll}{{\mathcal{L}}}_{\theta }({\bf{f}}\;)&=\mathop{\sum }\nolimits_{i = c}^{H-1}-\left[\,\text{log}\,{p}_{\theta }\left({\hat{{\bf{f}}}}_{i:H-1}| {{\bf{f}}}_{0:i-1},\{{{\bf{x}}}_{0:c-1},{{\bf{a}}}_{c-1:i-1}\}\right)\right]\end{array}$$

For more information about action-conditioned prediction models, see refs. ^35,43,44^. The LSTM classifier takes the predicted tactile frames and classifies each predicted time step as either slip or non-slip. The resulting slip signal, where the binary value of 0 indicates no slip and 1 indicates slip, is then used in the model predictive control framework to adjust the robot’s movements accordingly.

#### Slip classification model

To classify tactile frames as slip and non-slip signals, we leverage the temporal processing capabilities of LSTM networks, which have been shown to significantly enhance the classification performance by incorporating historical tactile features, compared with traditional classification methods such as support vector machines^45,46^.

Formally, we define the slip classification task as mapping the predicted tactile state sequence ${\hat{{\bf{f}}}}_{c:H-1}$ to a sequence of slip classifications ${\hat{{\bf{s}}}}_{c:H-1}$, where each ${\hat{{\bf{s}}}}_{i}\in \{{c}_{{\text{slip}}},{c}_{{\text{non-slip}}}\}$ denotes the slip status at time step *i* within the prediction window. The LSTM model is trained to learn the temporal dependencies in the tactile data, enabling it to effectively differentiate between slip and non-slip conditions.

Given a sequence of predicted tactile states ${\hat{{\bf{f}}}}_{c:H-1}$, the LSTM-based slip classifier processes these states sequentially, with the LSTM unit updates defined as follows:

6 $${{\bf{h}}}_{i},{{\bf{c}}}_{i}=\,\text{LSTM}\,\left({\hat{{\bf{f}}}}_{i},{{\bf{h}}}_{i-1},{{\bf{c}}}_{i-1}\right),\qquad i=c:H-1,$$

where **h**_*i*_ and **c**_*i*_ are the hidden and cell states at time step *i*, respectively. The output of the LSTM at each time step is passed through a fully connected layer to produce the slip classification logits, which are then converted into probabilities using a sigmoid activation function:

7 $${{\bf{s}}}_{i}=\sigma ({{\bf{W}}}_{s}{{\bf{h}}}_{i}+{{\bf{b}}}_{s}),\qquad i=c:H-1,$$

where *σ* is the sigmoid function, and **W**_*s*_ and **b**_*s*_ are the weight and bias of the output layer, respectively. The final slip classification ${\hat{{\bf{s}}}}_{i}$ is obtained by thresholding the probability output, assigning it to either the slip or non-slip class.

The architecture of the LSTM-based slip classification model is depicted in Fig. 2. Building on the work in ref. ^34^, which demonstrated that a simple LSTM-based tactile forward model combined with a slip classifier outperforms classifiers labelled with future slip instances, we use a state-of-the-art tactile forward model^35^ to estimate tactile states over the future prediction horizon and classify these predicted states accordingly.

To determine the stability of the object in the robot’s grip, the slip classification model primarily utilizes the shear force components from the predicted tactile states. To enhance the model’s generalization ability, we incorporate two dropout layers with a dropout probability of 0.5.

The slip classification dataset is imbalanced, comprising 16% slip instances and 84% non-slip instances. To address this imbalance, we train the classification model using the binary cross-entropy loss function, with a weighting scheme that penalizes slip instances more heavily than non-slip cases, using a relative weight of 3:1. This approach helps ensure that the classifier is conservative, with a strong tendency to predict actual slip instances as positive, leading to high recall rates, as reflected in the metrics in Supplementary Table 1 (right) for most of the train and test objects.

#### Slip control using trajectory modulation

Our proactive control approach, based on our previous work^34^, consists of a tactile forward model, a slip classifier and a predictive controller. A detailed presentation of model architecture and hyperparameters settings of ACTP and the slip classification models is included in Supplementary Section 1. We have made several improvements to the original approach that have led to substantial performance gains. First, we have incorporated our ACTP model to improve the accuracy of the slip classifier (Supplementary Table 1 provides the results). Second, we have extended the control variable from one DOF to six DOFs. This control strategy regulates the distance to the reference trajectory (which we call residual values), rather than learning the Cartesian velocity, as that in ref. ^47^. This results in improved optimization and better convergence and generalization. Additionally, we have expanded the object and trajectory sets, further improving the generalization of the approach. We denote the Cartesian-space velocity vector of the robot as $\overrightarrow{V}=({V}_{x},{V}_{y},{V}_{z},\,{W}_{x},{W}_{y},{W}_{z})$.

In the trajectory modulation loop, the first three components of the control vector represent the translational velocities along the Cartesian coordinate axes, whereas the last three components represent the angular velocities around those axes. Our goal is to minimize the future slip likelihood $L({s}_{c,\ldots ,H-1}| \alpha ,\beta ,{\bf{x}},{\bf{a}})=\mathop{\prod }\nolimits_{i = c}^{H-1}f({s}_{i}| \alpha ,\beta ,{{\bf{x}}}_{0:i},{{\bf{a}}}_{i})$ by learning the optimal deviation from the reference velocity profile, where *α*, *β*, **x** and **a** represent the tactile forward model and slip classifier parameters, past tactile states and planned robot actions, respectively. *s*_*i*_ indicates the *i*th observed slip value within a prediction horizon of length *c*. We choose spherical coordinates as the robot’s input {**a**_*c*−1_…**a**_*H*−2_} to adapt a given reference trajectory. As such, we modify the reference velocity vector by separately adjusting its norm and direction. To facilitate this modification, we represent the translational and angular velocities in spherical coordinates as follows.

$$\begin{array}{l}{\rho }_{v}=\sqrt{{V}_{x}^{2}+{V}_{y}^{2}+{V}_{z}^{2}};\,{\theta }_{v}={\tan }^{-1}\frac{{V}_{y}}{{V}_{x}};\,{\phi }_{v}={\cos }^{-1}\left(\frac{{V}_{z}}{\sqrt{{V}_{x}^{2}+{V}_{y}^{2}+{V}_{z}^{2}}}\right)\end{array}$$

8 $$\begin{array}{l}{\rho }_{w}=\sqrt{{W}_{x}^{2}+{W}_{y}^{2}+{W}_{z}^{2}};\,{\theta }_{w}={\tan }^{-1}\frac{{W}_{y}}{{W}_{x}};\,{\phi }_{w}={\cos }^{-1}\left(\frac{{W}_{z}}{\sqrt{{W}_{x}^{2}+{W}_{y}^{2}+{W}_{z}^{2}}}\right)\end{array}$$

**Extended Data Fig. 1:**
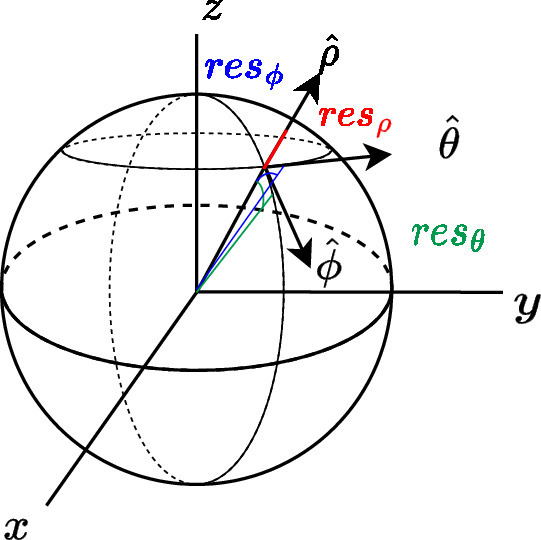
Residual Learning in spherical coordinate. Our trajectory modulation method learns residual to be added to the reference trajectory in spherical coordinate.

The variables *ρ*, *θ* and *ϕ* correspond to the norm, polar angle and azimuthal angle, respectively, in spherical coordinates. The relationship between the spherical and rectangular coordinates is illustrated in Extended Data Fig. 1. Using spherical coordinates enables the optimization process to separately learn the modifications needed for the norm and direction of the velocity vector in space. The optimization process returns the residual values, which are added to the components in equation (8) to modify the reference velocity profile.

9 $$\begin{array}{rcl}{\rho }_{v}^{* }&=&{\rho }_{v}+{\zeta }_{{\rho }_{v}};\,{\theta }_{v}^{* }={\theta }_{v}+{\zeta }_{{\theta }_{v}};\,{\phi }_{v}^{* }={\phi }_{v}+{\zeta }_{{\phi }_{v}}\\{\rho }_{w}^{* }&=&{\rho }_{w}+{\zeta }_{{\rho }_{w}};\,{\theta }_{w}^{* }={\theta }_{w}+{\zeta }_{{\theta }_{w}};\,{\phi }_{w}^{* }={\phi }_{w}+{\zeta }_{{\phi }_{w}}\end{array}$$

We express the residual values of equation (9) in the matrix form as ${\zeta }^{{\rm{d}}}=({\zeta }_{{\rho }_{v}},{\zeta }_{{\theta }_{v}},{\zeta }_{{\phi }_{v}},{\zeta }_{{\rho }_{w}},{\zeta }_{{\theta }_{w}},{\zeta }_{{\phi }_{w}})$. The residual trajectory values form the difference between the executed robot trajectory *ζ*^e^ and the reference trajectory *ζ*^r^ are ${\zeta }_{c:H-1}^{\;{\rm{d}}}={\zeta }_{c:H-1}^{\;{\rm{e}}}-{\zeta }_{c:H-1}^{\;{\rm{r}}}$ and ${\zeta }_{c:H-1}^{{\rm{e}}}=\{{{\bf{a}}}_{c-1}\ldots {{\bf{a}}}_{H-2}\}$, where ${\bf{a}}\in {{\mathbb{R}}}^{6}$ and $\zeta \in {{\mathbb{R}}}^{6\times c}$.

#### Parameterized residual learning for trajectory adaptation

To optimize the robot’s trajectories over a future time horizon (that is, ${i}_{{\rm{p}}}\in \{c,\ldots H-1\}\in {{\mathbb{Z}}}^{c}$), we represent the residual values using a parametric action representation^48^ that includes a weight parameter matrix **w** to be computed by the optimizer using Gaussian basis functions^49^, which is denoted by *Φ*: ${\zeta }_{c:H-1}^{\;{\rm{d}}}={{\bf{w}}}^{{\rm{T}}}\times \varPhi$, where $\varPhi \in {{\mathbb{R}}}^{n\times c}$ and ${\bf{w}}\in {{\mathbb{R}}}^{c\times 6}$. For the sake of simplicity, we express *ζ*_*c*:*H*−1_ by *ζ*.

The parameterized action space representation reduces computation complexity compared with direct search in continuous action space^48^. The benefit is more effective when the action is optimized in a future time horizon rather than a single time step due to the fewer search parameters in the optimization problem. We close the loop for the slip prevention controller by solving the constraint optimization in equation (10).

10 $$\begin{array}{r}\mathop{{\rm{arg}}\,{\rm{min}}}\limits_{{\bf{w}}}\,{\parallel {\zeta }^{{\rm{d}}}({\bf{w}},\varPhi )\parallel }_{2}\\ \,\text{Subject to}\,\,\,{{\mathbb{E}}}_{{{\bf{f}}}_{c:H-1}| ({{\bf{f}}}_{0:c-1},{\zeta }_{c:H-1}^{\;{\rm{e}}},{\zeta }_{0:c}^{\;{\rm{e}}})}[{s}_{c:H-1}({\zeta }^{{\rm{e}}}({\bf{w}},\varPhi ))]\,=\,0 \\ \,\,lb < {\dot{X}}_{i+1}-{\dot{X}}_{i}^{{\rm{obs}}} < ub\qquad \end{array},$$

where *ζ*^e^ = *ζ*^d^(**w**, *Φ*) + *ζ*^r^, and **f** denotes the tactile state vector $\in {{\mathbb{R}}}^{48\times c}$. The first nonlinear constraint in the optimization formulation is based on the expected value of the slip signal over the prediction horizon *c*, given by ${\mathbb{E}}[{s}_{c:H-1}({\zeta }^{{\rm{e}}}({\bf{w}},\varPhi ))]$, where *s* is a function of the robot’s future trajectory. The second constraint limits the difference between the generated robot velocities and the observed velocity (measurement) to ensure compliance with the robot’s low-level controller maximum acceleration limit. The optimization problem seeks to minimize the expected slip over the prediction horizon and remaining close to the provided reference trajectory.

The resulting spherical velocity components are transformed back to rectangular coordinates before being sent to the robot’s Cartesian-velocity controller as per equation (11). The *V*_*x*_, *V*_*y*_, *V*_*z*_, *W*_*x*_, *W*_*y*_ and *W*_*z*_ values are used to update the robot’s Cartesian-velocity controller, which regulates the robot’s motion along the desired trajectory and avoiding slipping.

$${V}_{x}={\rho }_{v}\,\sin ({\phi }_{v})\,\cos ({\theta }_{v}),\,{V}_{y}={\rho }_{v}\,\sin ({\phi }_{v})\,\sin ({\theta }_{v}),\,{V}_{z}={\rho }_{v}\,\cos ({\phi }_{v}),$$

11 $${W}_{x}={\rho }_{w}\,\sin ({\phi }_{w})\,\cos ({\theta }_{w}),\,{W}_{y}={\rho }_{w}\,\sin ({\phi }_{w})\,\sin ({\theta }_{w}),\,{W}_{z}={\rho }_{w}\,\cos ({\phi }_{w}).$$

We provide a detailed presentation of the grip force control method as the baseline controller for benchmarking our proactive controller in the Supplementary Information.

#### Training and testing

We trained our ACTP model and slip classification model offline using a dataset of pick-and-move tasks. Figure 2 illustrates the forward model and its architecture within a predictive control pipeline^50^.

The manipulation dataset consists of 420,000 data samples collected from 600 manipulation trials involving 13 box-shaped objects. Each object (including both train and test set objects; Supplementary Tables 1 and 2 list the dataset details) was involved in an equal number of trials, with three objects reserved as test objects, which were not seen during training. The performance of the ACTP and slip classification models was validated by analysing the mean absolute error and F-score values for the unseen objects, respectively. All models were trained on a Ubuntu machine equipped with an AMD Ryzen Threadripper CPU, NVIDIA GeForce RTX 2080 GPU and 64 GB of memory. The training was conducted using the PyTorch v. 1.13.1 library with CUDA v. 11.7. The trained ACTP and slip classification models are then used with fixed-weight parameters for real-time control tests.

During testing, the trajectory modulation module utilizes the inference from these two models in an online optimization loop to determine the optimal next robot action that minimizes the likelihood of slip occurrence in a receding horizon framework. We will now present the design details for each building block of the proactive control system (Fig. 2).

#### Objects, metrics and comparison method

Table 2 presents the objects used for training and testing our controller (Supplementary Table 1 provides the pictures of each object). It also details the performance metrics for both train and test objects. The train and test sets are based on the data collection for training the underlying tactile forward model and slip classifier in the proactive control, consisting of ten train objects and three test objects. The performance metrics for the slip controller include rotation of >6°, time steps (RTS) and maximum object rotation (MOR) in degrees. RTS represents the number of time steps during which the object’s rotation exceeded the slip classification threshold (6°). Smaller RTS values indicate better slip avoidance performance. The proactive control achieved excellent performance with no slip instances (RTS = 0) for five objects in the train set and two objects in the test set (boldface values in the RTS column). The MOR values show that for five objects, the maximum rotation slightly exceeded 6°, the threshold set for slip classification. This may be attributed to the imprecision of the forward model, the classifier (Supplementary Table 1) or the threshold we set on the number of iterations in the controller computations to find the optimal actions (as we allow only ten optimizer iterations due to real-time constraints). Nonetheless, MOR remains below 9°, preventing failure despite slip instances. Comparing the mean values of MOR and RTS for the train and test sets demonstrates that our proposed controller generalizes well to unseen objects during training and effectively avoids slip instances on average. DRT denotes the distance to the reference trajectory, calculated by summing the Euclidean distance between the reference and optimized trajectories across all task time steps (measured in m s^−1^). ROV represents the resulted optimality value, indicating the convergence of the optimization process at the final iteration.

### Reporting summary

Further information on research design is available in the Nature Portfolio Reporting Summary linked to this article.

## Supplementary information

Supplementary Information Supplementary Sections 1 and 2 (explaining the ACTP model, training and test results) and Tables 1–4.

Reporting Summary

Supplementary Video 1 Proposed approach and robot performance.

## Figures and Tables

**Extended Data Fig. 2:**
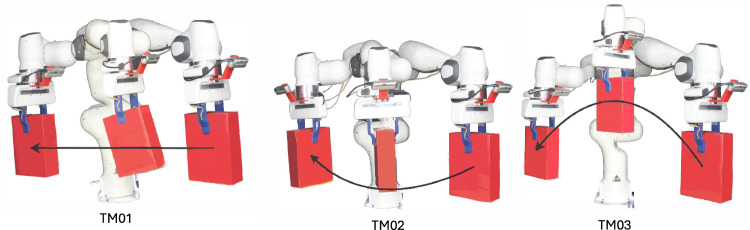
Task maneuvers. Task maneuvers in Table 1 and 3.

## Data Availability

The experimental data used in this work including the robotic experiments are available at https://proactive-control.github.io/.

## Code availability

The code used for training and testing the slip prediction models, as well as implementing the slip controller, is publicly available via GitHub at https://github.com/imanlab/bgf and via Zenodo at 10.5281/zenodo.14524629 (ref. ^51^).
