# Supplementary material for: Bioinspired trajectory modulation for effective slip control in robot manipulation
Source: Nat Mach Intell. 2025 Jul 22;7(7):1119–28. doi: 10.1038/s42256-025-01062-2 (PMC12283357; doi:10.1038/s42256-025-01062-2)
Supplement: Supplementary file 1 — Supplementary Sections 1 and 2 (explaining the ACTP model, training and test results) and Tables 1–4. [file 42256_2025_1062_MOESM1_ESM.pdf]

nature machine intelligence

Supplementary information https://doi.org/10.1038/s42256-025-01062-2

Bioinspired trajectory modulation for effective slip control in robot manipulation

In the format provided by the authors and unedited

Appendix Table of Contents

Contents

A1 Results of Data-driven Forward Models **2**

A2 Methodology **6**

A1 Results of Data-driven Forward Models

The Proactive Control benefits from an internal forward model for slip prediction. Our forward model consists of an Action-conditioned Tactile Prediction model (ACTP) [1] and a slip classifier [2] described below.

ACTP: Our proposed method predicts the future tactile state vector $x_{t:t+h}$ given a history of tactile readings $x_{t-c:t-1}$ (context) with c denoting the context length, a history of robot state vectors $r_{t-c:t-1}$, and a sequence of future robot state vectors $r_{t:t+h}$ where h denotes the prediction horizon length. We chose $c = 10$ and $h = 10$ based on computational complexity considerations and the controller's performance, as discussed in [1]. Fig. 1 displays the Shear X and Y, and Normal Z tactile readings for a taxel in the centre of the sensor, along with the $t + 1$, $t + 5$, and $t + 10$ predicted signals, for automated robot movements (top row) and kinaesthetic motions (bottom row) for a test object (Domino). The results demonstrate that our proposed method has high precision in predicting tactile signals.

Furthermore, as the prediction horizon increases, the distance between the predicted tactile signal and the true tactile signal also increases, resulting in weaker quantitative results (Tactile Prediction column in Table 1). However, by examining the qualitative results in Fig. 1, we can observe changes in the tactile signals earlier, leading to better insights into the performance of the ACTP. Overall, our findings suggest that the ACTP provides accurate enough predictions of tactile signals for the test object in both kinaesthetic and automatic motion tasks.

The first two left columns of Table 1 present the quantitative tactile prediction analysis with Mean Absolute Error (MAE) and Mean Squared Error (MSE) for each train and test object, highlighting the impact of objects' variance in friction, weight, and size on tactile prediction results. The reported metrics for the training objects, which were seen during the model training, reflect the performance on the holdout set from the training data. For the test objects, which

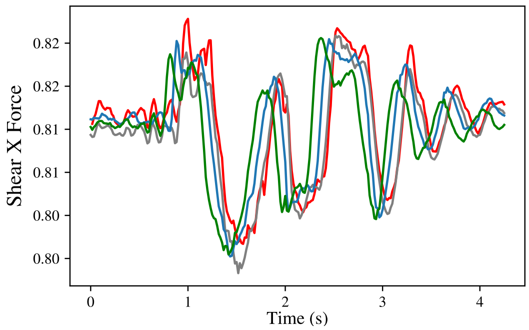

(a) Shear X vs time

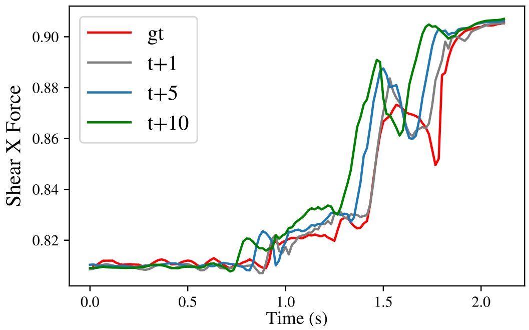

(b) Shear X vs time

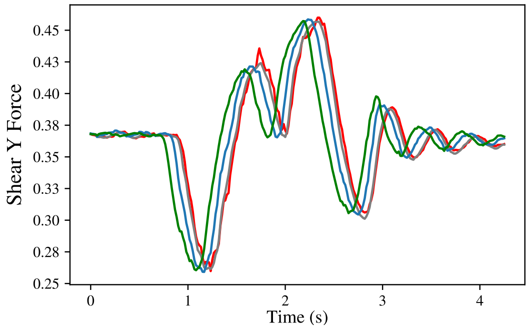

(c) Shear Y vs time

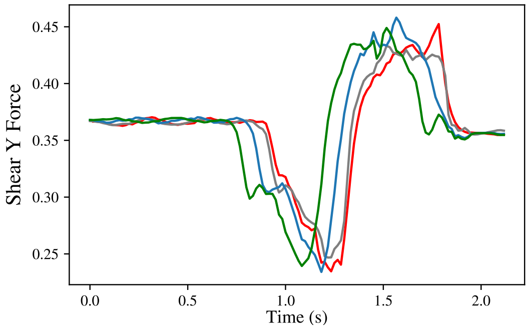

(d) Shear Y vs time

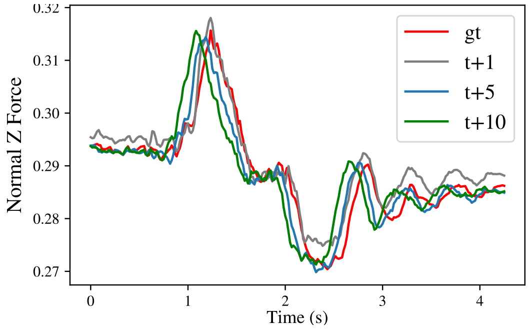

(e) Normal Z vs time

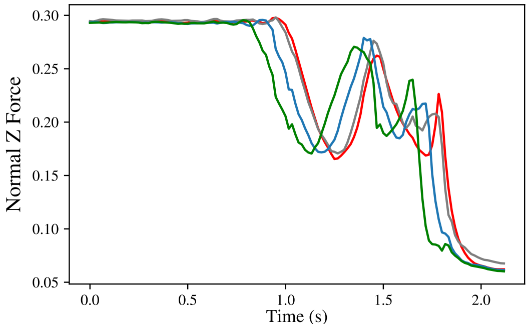

(f) Normal Z vs time

Figure 1: Tactile signal prediction for a sample taxel (third row and second column from top left) in x, y, and z directions in an automatic robot motion (left column) and a kinaesthetic motion task (right column). Tactile force readings have been normalized to a 0–1 range across the entire training dataset.The prediction window of the forward model is $t+1 : t+10$ and we show three prediction time steps (e.g. $t+1$, $t+5$, and $t+10$) beside the ground truth (gt) signal in the graphs. The force values are normalised to be between 0-1 and the horizontal axis shows task time (s).

were not seen during training, the metrics encompass the performance across all data points. The MSE values align with the MAE values. CUPa SOUP and CORNFLOUR are the easiest

and most difficult objects within the train set for tactile prediction. The Mean row displays the mean and standard deviation of the errors among the train objects. Results for the test objects demonstrate that the model generalises well to unseen objects, with MAE and MSE mean values between the train and test objects relatively close to each other.

Slip Classification: The slip classification module maps the output of the ACTP to binary slip signals indicating whether an object is stable or not. Minimising the slip signal likelihood maximises the object's stability. Because we have an imbalanced classification problem, we use precision, recall, and F-score to evaluate the classifier's performance besides accuracy. Precision measures the proportion of predicted slip values that were actually slip instances, while recall measures the proportion of actual slip instances that were correctly identified by the classifier. The F-score is the weighted mean of precision and recall[1]respectively:

$$Accuracy = \frac{TP + TN}{TP + FP + TN + FN},$$

$$Precision = \frac{TP}{TP + FP},$$

$$Recall = \frac{TP}{TP + FN},$$

$$f\_score = 2 \times (precision * recall)/(precision + recall).$$

We present the mean classification metrics values over the prediction horizon.

Table 1 (right) shows that the performance of the classification module can be independent of the accuracy of the prediction module. For instance, the CORNFLOUR object has smaller tactile prediction errors than the Ravya THINS object, but the classification performs better on Ravya THINS. The mean classification metrics for the train and test objects indicate that the full slip prediction model can generalise to unseen objects. However, the variance in the classification performance can also be related to the number of slip instances for each object.

[1] slip and non-slip classes are denoted by positive (P) and negative (N) classes and true and false predictions are shown by T and F

Table 1: Tactile prediction (left) and slip classification (right) performance on train and test objects. ↓ or ↑ show lower or higher values are better, respectively. The Mean Absolute Error (MAE) and Mean Squared Error (MSE) values are calculated for normalised tactile force values. The boldface values show the best scores for the train and test set in each column.

Object			**Tactile Prediction**		**Slip Classification**			
			MAE$\times 10^2$ ↓	MSE$\times 10^3$ ↓	Accuracy ↑	Precision ↑	Recall ↑	F-score ↑
Train	01	BreadSticks	0.64 ± 0.05	0.14 ± 0.01	0.77 ± 0.01	0.60 ± 0.02	0.99 ± 0.00	0.75 ± 0.01
	02	CORNFLOUR	**0.37 ± 0.02**	**0.03 ± 0.00**	0.64 ± 0.02	0.48 ± 0.03	0.99 ± 0.01	0.65 ± 0.02
	03	Ravyta THINS	0.50 ± 0.03	0.06 ± 0.00	0.95 ± 0.01	0.95 ± 0.02	0.99 ± 0.02	**0.97 ± 0.01**
	04	PUFF pastry	0.50 ± 0.01	0.06 ± 0.02	0.86 ± 0.02	0.83 ± 0.04	0.96 ± 0.02	0.89 ± 0.02
	05	GRISSINI	0.60 ± 0.05	0.10 ± 0.02	0.62 ± 0.03	0.59 ± 0.02	0.99 ± 0.00	0.71 ± 0.01
	06	Batter Mix	0.47 ± 0.03	0.06 ± 0.01	**0.96 ± 0.01**	**0.95 ± 0.02**	0.99 ± 0.01	**0.97 ± 0.02**
	07	Jaffa cake	0.65 ± 0.03	0.12 ± 0.02	0.81 ± 0.01	0.77 ± 0.01	0.99 ± 0.01	0.87 ± 0.02
	08	KLEENEX	0.56 ± 0.04	0.08 ± 0.03	0.80 ± 0.03	0.76 ± 0.03	**1.0 ± 0.00**	0.86 ± 0.04
	09	CHEEZEIT	0.59 ± 0.03	0.10 ± 0.02	0.92 ± 0.02	0.91 ± 0.02	0.99 ± 0.01	0.95 ± 0.01
	10	CUPa SOUP	1.29 ± 0.12	0.86 ± 0.05	0.76 ± 0.01	0.75 ± 0.02	0.94 ± 0.02	0.83 ± 0.02
	Mean		0.61 ± 0.23	0.16 ± 0.21	0.80 ± 0.11	0.75 ± 0.15	0.98 ± 0.01	0.84 ± 0.10
	Nazari et al. [2]		-	-	0.69 ± 0.03	0.61 ± 0.04	0.95 ± 0.02	0.74 ± 0.03
Test	11	RICE	0.71 ± 0.04	0.21 ± 0.04	0.69 ± 0.05	0.57 ± 0.03	**0.98 ± 0.01**	0.69 ± 0.02
	12	Carrs	0.62 ± 0.02	0.11 ± 0.01	0.71 ± 0.02	0.61 ± 0.03	0.97 ± 0.00	0.79 ± 0.01
	13	Domino	**0.56 ± 0.09**	**0.08 ± 0.01**	**0.94 ± 0.01**	**0.94 ± 0.02**	0.96 ± 0.02	**0.95 ± 0.02**
	Mean		0.63 ± 0.06	0.13 ± 0.05	0.78 ± 0.11	0.70 ± 0.16	0.97 ± 0.00	0.81 ± 0.10
	Nazari et al. [2]		-	-	0.63 ± 0.02	0.59 ± 0.04	0.90 ± 0.01	0.71 ± 0.01

In the data collection phase, the human operating the kinaesthetic data collection attempted to generate motions that lead to a uniform number of slip instances for different objects, but differences in friction and weight can result in varying numbers of slip instances across objects. The slip classification accuracy ranges from 0.62 to 0.96, precision ranges from 0.48 to 0.95, recall ranges from 0.94 to 1.0, and F-score ranges from 0.71 to 0.97.

We conducted a comparison of slip classification performance using our proposed approach, which incorporates a state-of-the-art tactile forward model, with the approach used by Nazari et al. [2] (see last rows in Table 1 (right)). Our results show significant improvement in all metrics for both the train and test object sets. This improvement is attributed to the inclusion of the forward model, which allows for the classification to be applied on the estimated future tactile states instead of relying solely on past tactile measurements as in [2]. This change results in a more straightforward mapping for the classifier module, leading to improved slip classification performance as further evidence of the effectiveness of utilising the tactile forward model in the

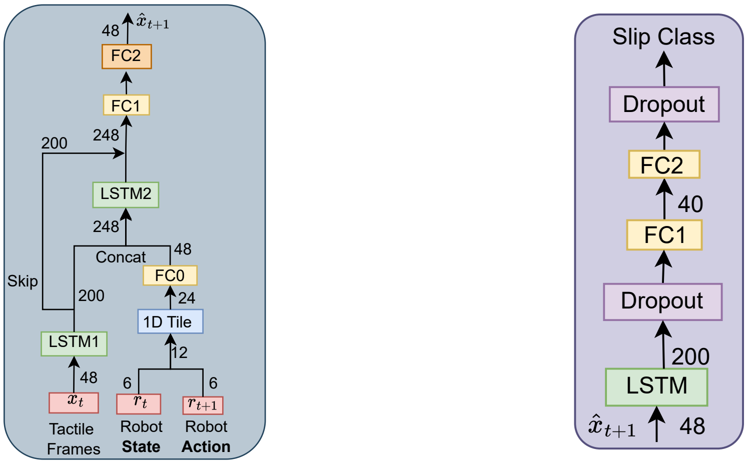

(a) (b)

Figure 2: A single forward propagation path of (a) Action-conditioned tactile prediction model. (b) Slip classification model.

slip control method as stated in our third hypothesis.

A2 Methodology

In our setup, each tactile reading $\mathbf{x} \in \mathbb{R}^{48}$ corresponds to the uSkin sensor readings for 16 taxels, and each robot action $\mathbf{r} \in \mathbb{R}^{6}$ represents the robot's task space velocity in the base frame. We designed the model architecture (see Fig. 2(a)) based on the state-of-the-art tactile prediction models [1] and used Mean Absolute Error (MAE) and Mean Squared Error (MSE) loss functions during training. We found that MAE resulted in more stable training overall.

Grip Force Modulation for Slip Control: The baseline grip force controller was implemented using a SMC Z gripper, which supports real-time position control—a necessary adaptation given the hardware limitations of the Franka Emika robot's default gripper, which does not allow real-time control due to its blocking TCP communication. The grip force was adjusted by modulating the finger positions in discrete steps. Through empirical tuning, we identified a

4 mm step size as the optimal setting. This size provided a sufficient increase in grip force while minimising the risk of excessive object deformation. Larger step sizes caused undesirable deformation, while smaller steps resulted in a slower force increase and a less responsive control strategy. The 4 mm step size was, therefore selected based on the stiffness characteristics of the objects in our test set, ensuring effective and responsive grip force control. 4 mm step is optimal for minimizing deformation and ensuring an effective response time. The reactive system, as a baseline slip controller for comparison, is based on a slip detection model which uses the recent history of tactile readings $\mathbf{x}_{t-c:t-1}$ for slip classification at time t. While the classification model is the same as the trajectory modulation method (shown in Fig 2 (b)), the input features are uSkin measurements as opposed to tactile predictions in the forward model in the trajectory modulation controller. Every time the predicted slip signal S_i is positive, the SMC gripper tightens its grip by reducing the grasp width by 4 mm. The formulation of the grip force modulation for slip control is defined as:

$$G_{i+1} = \begin{cases} G_i - \delta, & \text{if } S_i = 1 \\ G_i, & otherwise \end{cases}$$

Where G denotes the grip width and δ is the adjustment width.

Robotic Manipulation Dataset: The objective of our dataset collection is to encompass a diverse range of robot motion profiles within the task space, including various velocities and motion types (e.g., linear, and rotational). We also aim to include a sufficient number of positive slip instances for training our slip classification models and to account for different frictional behaviors of the objects used. To achieve this, we collected data using two primary robot motion policies: (i) kinaesthetic teaching, where a human operator guides the robot through various motion classes, and (ii) automated motion generated by the Franka robot's Cartesian velocity controller, which produces predefined reference trajectories. The object sets were selected to provide a wide range of variations in friction coefficient, weight, contact geometry, size, rigidity,

Table 2: Overview of the objects utilized for training and testing the Action Conditioned Tactile Prediction (ACTP) and slip classification models. The table lists the first 10 objects used in the training set and the subsequent 4 objects reserved for the test set. After the training phase, these models were further evaluated through real-time performance tests with the proactive controller to assess their robustness and accuracy under dynamic conditions.

01	02	03	04	05	06	07
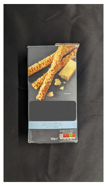	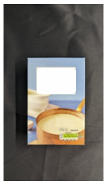	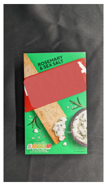	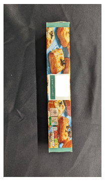	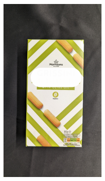	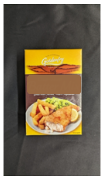	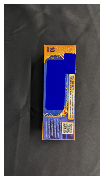
08	09	10	11	12	13	14
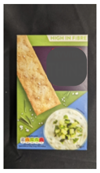	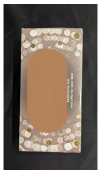	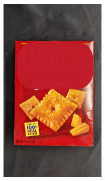	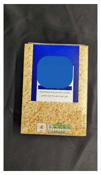	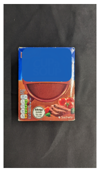	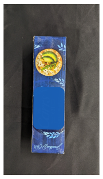	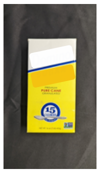

and dynamic weight.

Due to the low spatial resolution of the uSkin taxel, predicting tactile interactions for objects with varying contact geometries and rigidities presents challenges, as discussed in [2]. Future research could explore the generalization of the ACTP model using high-resolution tactile sensors to address these challenges and improve performance with previously unseen geometries.

The dataset includes three types of data: tactile data, robot state data, and slip signals. Tactile data is recorded as a 48-dimensional vector (3 forces for each of the 16 taxels) at a sampling rate of 100 Hz. Robot state data is recorded as a 6-dimensional vector (Cartesian velocity) at 1000 Hz. Slip signals are binary, sampled at 60 Hz, and are derived from post-processing ArUco marker data attached to the object. All data types are synchronized at 60 Hz using the ROS *ApproximateTime* policy. The multimodal deep neural network, which integrates tactile and robot state data to predict binary slip events, requires synchronized data from all sensing

Object id		Number of samples in the dataset for training and testing in training and testing of ACTP and SCM		
		kinaesthetic Teaching	Automatic motion with predefined ref trajectory	Sum
**Train Set**	1	30	15	45
	2	30	15	45
	3	30	15	45
	4	30	15	45
	5	30	15	45
	6	30	15	45
	7	30	15	45
	8	30	15	45
	9	30	15	45
	10	30	15	45
**Test Set**	1	30	15	45
	2	30	15	45
	3	30	15	45
**Sum**				**600**

Table 3: This table presents the number of manipulation trials used for training and testing the action-conditioned tactile prediction (ACTP) and slip classification models (SCM). The results of these models are presented in Table 1 of this Appendix.

modalities for both model training and deployment.

We present the full set of objects used in our experiments in Table 2. The dataset consists of 10 objects designated for training the tactile prediction and slip classification models, while an additional 4 objects were reserved for testing. These objects were selected to represent a diverse range of friction, weight, and material properties, ensuring robust model generalisation. After training, the performances of the models were evaluated through real-time slip controller tests, validating their efficacy in dynamic manipulation tasks. The object set used in this study consists of box-shaped objects with flat contact geometries. This selection was due to the limited spatial resolution of the uSkin tactile sensor, which made it impractical to include more complex contact geometries in the dataset. A separate study in [3] further explored the generalisation ca-

Object id		Predefined reference trajectories										
		TM-1			TM-2			TM-3				
		trap	cub	qui	trap	cub	qui	trap	cub	qui	Novel start pose	Sum
**Test Set**	1	10	10	10	10	10	10	10	10	10	90	180
	2	10	10	10	10	10	10	10	10	10	90	180
	3	10	10	10	10	10	10	10	10	10	90	180
**Sum**												**540**

Table 4: Number of manipulation trials conducted to test the slip controller. For definitions of Test Motion (TM) 1, 2, and 3, please refer to Table 1 in the main manuscript. Each test motion is evaluated using three different reference trajectories: trapezoidal, cubic, and quintic. The results presented in Table 3 of the main manuscript correspond to trials performed with the ‘‘Novel Start Pose’ for these trajectories.

pabilities of the ACTP model with a larger object set size. In this work, our primary focus is on evaluating the slip controller's performance, rather than on the deep models' generalisation to diverse contact topologies.

Our experiment consisted of two main stages: Stage 1, which focused on training and testing the action-conditioned tactile prediction and slip classification models, and Stage 2, which tested the performance of the slip prevention controller. A total of 600 trials were exclusively collected for Stage 1, with the data evenly distributed across 13 objects, averaging approximately 45 trials per object. Among these, the data from 3 test objects were specifically reserved for evaluating the tactile prediction and slip classification models (Figure 3).

Stage 2 trials were conducted to assess the slip controller's performance with the test objects in real-time robot experiments, as shown in Table 2 of the main manuscript. These trials are independent of the 600 trials used in Stage 1 for offline training and testing of the tactile prediction and slip classification models. The number of trials for both stages is detailed in Table 3 and 4.

Details and results of the human subject tests, Which shed light on how humans control object slip when grip force control is insufficient, are provided in [4].

References

[1] W. Mandil, K. Nazari, A. G. Esfahani, *Action Conditioned Tactile Prediction: a case study on slip prediction*, *Robotics: Science and Systems* (2022).

[2] K. Nazari, W. Mandil, A. G. Esfahani, *Proactive slip control by learned slip model and trajectory adaptation*, *Conference on Robot Learning*, 751–761 (2023).

[3] W. Mandil, A. Ghalamzan-E, *Combining vision and tactile sensation for video prediction*, *arXiv preprint arXiv:2304.11193*, (2023).

[4] K. Nazari, W. Mandil, M. Santello, A. Ghalamzan, *Human Slip Control: Investigating the Role of Hand Acceleration Modulation in Preventing Slips*, *BioRxiv preprint doi.org/10.1101/2024.12.29.630651, (2024).*
