# Supplementary material for: Bioinspired trajectory modulation for effective slip control in robot manipulation
Source: Nat Mach Intell. 2025 Jul 22;7(7):1119–28. doi: 10.1038/s42256-025-01062-2 (PMC12283357; doi:10.1038/s42256-025-01062-2)
Supplement: Supplementary file 2 — Reporting Summary [file 42256_2025_1062_MOESM2_ESM.pdf]

nature portfolio

Corresponding author(s): NATMACHINTELL-A24010109C

Last updated by author(s): 14/03/2025

Reporting Summary

Nature Portfolio wishes to improve the reproducibility of the work that we publish. This form provides structure for consistency and transparency in reporting. For further information on Nature Portfolio policies, see our Editorial Policies and the Editorial Policy Checklist.

Statistics

For all statistical analyses, confirm that the following items are present in the figure legend, table legend, main text, or Methods section.

n/a	Confirmed	
☐	☒	The exact sample size (*n*) for each experimental group/condition, given as a discrete number and unit of measurement
☐	☒	A statement on whether measurements were taken from distinct samples or whether the same sample was measured repeatedly
☐	☒	The statistical test(s) used AND whether they are one- or two-sided<br>*Only common tests should be described solely by name; describe more complex techniques in the Methods section.*
☒	☐	A description of all covariates tested
☒	☐	A description of any assumptions or corrections, such as tests of normality and adjustment for multiple comparisons
☐	☒	A full description of the statistical parameters including central tendency (e.g. means) or other basic estimates (e.g. regression coefficient) AND variation (e.g. standard deviation) or associated estimates of uncertainty (e.g. confidence intervals)
☒	☐	For null hypothesis testing, the test statistic (e.g. *F*, *t*, *r*) with confidence intervals, effect sizes, degrees of freedom and *P* value noted<br>*Give P values as exact values whenever suitable.*
☒	☐	For Bayesian analysis, information on the choice of priors and Markov chain Monte Carlo settings
☒	☐	For hierarchical and complex designs, identification of the appropriate level for tests and full reporting of outcomes
☒	☐	Estimates of effect sizes (e.g. Cohen's *d*, Pearson's *r*), indicating how they were calculated

Our web collection on statistics for biologists contains articles on many of the points above.

Software and code

Policy information about availability of computer code

Data collection	all the software and codes for data collection are available in public GitHub repository https://github.com/imanlab/bgf and https://zenodo.org/records/14524629
Data analysis	All the software and codes for data analysis are available in public GitHub repository https://github.com/imanlab/bgf and https://zenodo.org/records/14524629. We used Ubntu 20.04 LTS, CUDA 11.7, ROS Noetic and Pytorch Version 1.13.1

For manuscripts utilizing custom algorithms or software that are central to the research but not yet described in published literature, software must be made available to editors and reviewers. We strongly encourage code deposition in a community repository (e.g. GitHub). See the Nature Portfolio guidelines for submitting code & software for further information.

Data

Policy information about availability of data

All manuscripts must include a data availability statement. This statement should provide the following information, where applicable:

- Accession codes, unique identifiers, or web links for publicly available datasets
- A description of any restrictions on data availability
- For clinical datasets or third party data, please ensure that the statement adheres to our policy

All the data for robot (and human) experimetns are publicly available on paper website: https://proactive-control.github.io/

Research involving human participants, their data, or biological material

Policy information about studies with human participants or human data. See also policy information about sex, gender (identity/presentation), and sexual orientation and race, ethnicity and racism.

Reporting on sex and gender	N/A
Reporting on race, ethnicity, or other socially relevant groupings	N/A
Population characteristics	N/A
Recruitment	N/A
Ethics oversight	N/A

Note that full information on the approval of the study protocol must also be provided in the manuscript.

Field-specific reporting

Please select the one below that is the best fit for your research. If you are not sure, read the appropriate sections before making your selection.

☒ Life sciences ☐ Behavioural & social sciences ☐ Ecological, evolutionary & environmental sciences

For a reference copy of the document with all sections, see nature.com/documents/nr-reporting-summary-flat.pdf

Life sciences study design

All studies must disclose on these points even when the disclosure is negative.

Sample size	Our training and testing datasets consist of 600 and 540 pick-and-place manipulation trials, respectively. The dataset sizes were determined based on findings from a preliminary study (Nazari et al., 2022), while also considering the computational constraints of our neural network and hardware capabilities. This selection ensures sufficient variation in contact states across different trials, enabling robust learning and generalization.
Data exclusions	No data were excluded from the analysis.
Replication	The public GitHub repository of the project provides comprehensive, step-by-step instructions for replicating the experiments, ensuring reproducibility and ease of implementation.
Randomization	Randomization was not applicable, as the dataset was intentionally structured to maintain a balanced representation of contact states, ensuring the model effectively learns slip dynamics.
Blinding	Blinding was not applicable, as data collection and labeling were based on direct sensor measurements, thereby eliminating any potential for subjective bias.

Reporting for specific materials, systems and methods

We require information from authors about some types of materials, experimental systems and methods used in many studies. Here, indicate whether each material, system or method listed is relevant to your study. If you are not sure if a list item applies to your research, read the appropriate section before selecting a response.

Materials & experimental systems

n/a	Involved in the study	
☒	☐	Antibodies
☒	☐	Eukaryotic cell lines
☒	☐	Palaeontology and archaeology
☒	☐	Animals and other organisms
☒	☐	Clinical data
☒	☐	Dual use research of concern
☒	☐	Plants

Methods

n/a	Involved in the study	
☒	☐	ChIP-seq
☒	☐	Flow cytometry
☒	☐	MRI-based neuroimaging

Plants

Seed stocks	N/A
Novel plant genotypes	N/A
Authentication	N/A
